# Same-session endoscopic ultrasound-guided drainage and closure of a challenging peritoneal pseudocyst in a high-surgical-risk patient

**DOI:** 10.1055/a-2719-8306

**Published:** 2025-11-04

**Authors:** Oscar Victor Hernández Mondragón, Oscar Omar Lopez Padilla, Kevin Josue Pintor Belmontes

**Affiliations:** 1 37767 Division of Endoscopy, Specialties Hospital, National Medical Center Century XXI, Mexico City, Mexico

Peritoneal pseudocyst (PP) is a rare complication in patients with end-stage renal disease (ESRD) undergoing current or prior peritoneal dialysis (PD) 1 2 3 . In symptomatic cases, surgery is the standard treatment; however, in high-surgical-risk patients or anatomically complex PP, neither surgery nor percutaneous drainage may be feasible 4 5 . We report the case of a PP in a high-surgical-risk patient, successfully managed with endoscopic ultrasound (EUS)-guided drainage and same-session closure using a lumen-apposing metal stent (LAMS).

A 35-year-old woman with ESRD and multiple comorbidities, previously on PD for 1 year prior, presented with abdominal pain, nausea, vomiting, and intolerance to oral intake. Abdominal computed tomography (CT) revealed a 25-cm encapsulated, nonseptated, well-defined fluid collection compressing the stomach ( Fig. 1 ). Due to her clinical condition and anatomical location, she was not a candidate for surgical or percutaneous treatment. After discussing risks and benefits, EUS-LAMS drainage was performed. EUS confirmed the PP ( Fig. 2 ); a 20 mm × 10 mm hot AXIOS stent (Boston Scientific, Marlborough, MA, USA) was placed, achieving drainage of clear fluid. Balloon dilation up to 15 mm (CRE 12–15 mm, Boston Scientific, Marlborough, MA, USA) was performed. Upper endoscopy confirmed the absence of septations and allowed complete drainage ( Fig. 3 ). The LAMS was removed, and gastric closure was achieved with Mantis and Resolution clips (Boston Scientific, Marlborough, MA, USA) ( Video 1 ). The patient experienced full clinical improvement. Follow-up CT at 24 hours showed a small residual collection and pneumoperitoneum, with resolution of gastric compression. Oral intake was resumed, and she was discharged on day 2. At the 3-month follow-up, no recurrence was observed.

**Fig. 1:**
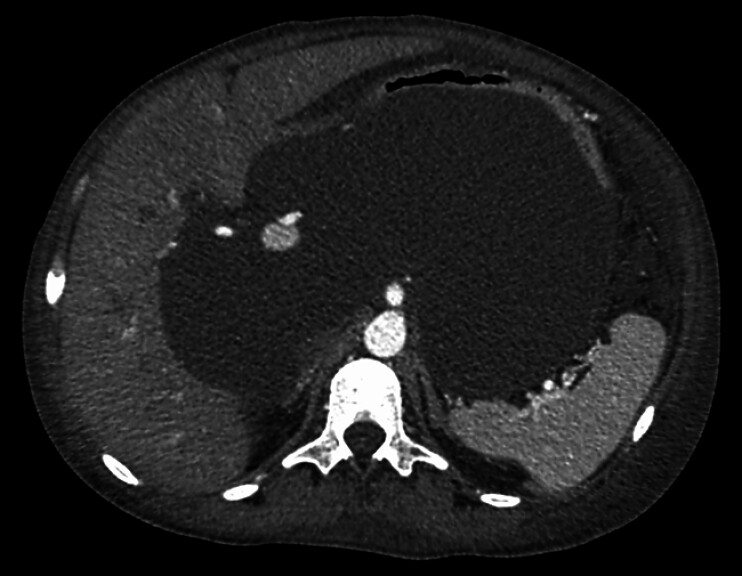
Initial abdominal CT scan shows a well-defined, encapsulated peritoneal pseudocyst in the lesser sac, causing compression of the posterior gastric wall.

**Fig. 2:**
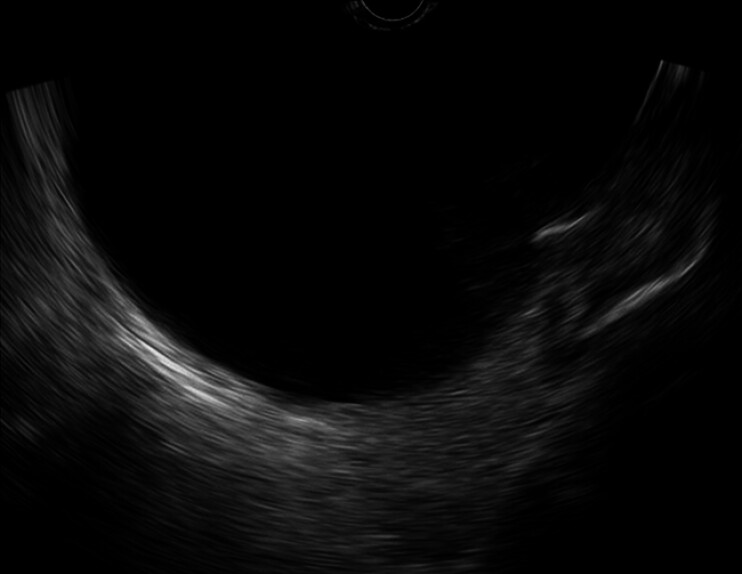
Endoscopic ultrasound image confirms a very large nonseptated, well-defined fluid collection.

**Fig. 3:**
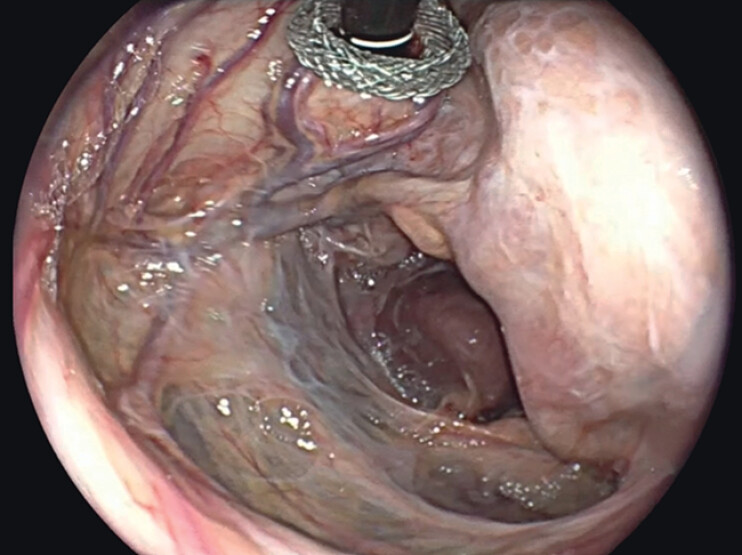
Direct endoscopic view of the peritoneal pseudocyst through the lumen-apposing metal stent, revealing the lesser sac cavity.

EUS-guided drainage of a large peritoneal pseudocyst with lumen-apposing metal stent, direct endoscopic drainage, and finally, gastric closure with through-the-scope clips.Video 1

To our knowledge, this is the first report of a PP successfully treated with same-session EUS-LAMS drainage and closure, highlighting the feasibility and safety of this minimally invasive option in high-surgical-risk or anatomically challenging cases.

Endoscopy_UCTN_Code_TTT_1AS_2AC

## Notes

Endoscopy E-Videos https://eref.thieme.de/e-videos
*E-Videos* is an open access online section of the journal *Endoscopy* , reporting on interesting cases and new techniques in gastroenterological endoscopy. All papers include a high-quality video and are published with a Creative Commons CC-BY license. Endoscopy E-Videos qualify for HINARI discounts and waivers and eligibility is automatically checked during the submission process. We grant 100% waivers to articles whose corresponding authors are based in Group A countries and 50% waivers to those who are based in Group B countries as classified by Research4Life (see: https://www.research4life.org/access/eligibility/ ). This section has its own submission website at https://mc.manuscriptcentral.com/e-videos .
